# Adaptability of Wild-Growing Tulips of Greece: Uncovering Relationships between Soil Properties, Rhizosphere Fungal Morphotypes and Nutrient Content Profiles

**DOI:** 10.3390/biology12040605

**Published:** 2023-04-16

**Authors:** Fotis Bilias, Anastasia-Garyfallia Karagianni, Ioannis Ipsilantis, Ioulietta Samartza, Nikos Krigas, Georgios Tsoktouridis, Theodora Matsi

**Affiliations:** 1 Soil Science Laboratory, School of Agriculture, Aristotle University of Thessaloniki, 54124 Thessaloniki, Greece; 2 Institute of Plant Breeding and Genetic Resources, Hellenic Agricultural Organization Demeter, P.O. Box 60458, 57001 Thessaloniki, Greece; 3 Theofrastos Fertilizers, Irinis & Filias, Examilia Korithias, 20100 Korinthos, Greece

**Keywords:** *Tulipa* spp., elemental variability, plant nutrients, phytogeographical units, edaphic variations, arbuscular mycorrhizal fungi

## Abstract

**Simple Summary:**

Greek wild-growing tulips are protected plants, about which there is scarce knowledge regarding their natural nutrient status and rhizosphere fungal morphotypes. In this study, we collected plant (above-ground and bulb material) and soil samples from 13 tulip species across three phytogeographical units in Greece, and we assessed the tulips’ nutrient content and soil properties to determine their interrelationships. We found that soil variables significantly influenced tulip nutrient content, with up to 67% of the detected variability explained by soil properties. Correlation patterns were also found between tulips’ essential nutrients. Our study revealed clear distinctions in nutrient content among tulip species from different spatial (phytogeographic) units. The findings shed light on Greek tulips’ adaptability and resilience in their natural habitats and may facilitate their domestication in artificial settings.

**Abstract:**

Wild-growing Greek tulips are protected plants but almost nothing is known about their natural nutrient status and rhizosphere fungal morphotypes in the wild, thus no insight is currently available into their growth and adaptation to their natural environment or artificial settings. To this end, several botanical expeditions were conducted with a special collection permit, and 34 tulip and soil samples were collected, representing 13 species from two phytogeographical regions of Greece (North Aegean Islands, Crete Island) and seven regions of mainland Greece. The tulips’ content in essential macro- and micro-nutrients, respective physicochemical soil properties, and rhizosphere fungal morphotypes were assessed across samples, and all parameters were subjected to appropriate statistical analysis to determine their interrelationships. The results showed that soil variables played a significant role in shaping tulips’ nutrient content, explaining up to 67% of the detected variability as in the case of phosphorus (P) in the above-ground plant tissue. In addition, significant correlations were observed (with an *r* value of up to 0.65, *p <* 0.001) between essential nutrients in the tulips, such as calcium (Ca) and boron (B). The principal component analysis (PCA) revealed that between the three spatial units examined, the total variability of tulips’ nutrient content produced a clear distinction among sampled species, while the first two PCA axes managed to explain 44.3% of it. This was further confirmed by the analysis of variance (ANOVA) results which showed corresponding significant differences (at *p* < 0.05) in both the tulips’ nutrient content and the studied soil properties as well (mean values of N, P, and K in the North Aegean Islands tulips’ nutrient content, up to 53%, 119%, and 54% higher compared to those of the Crete Island, respectively). Our study sheds light on Greek tulips’ adaptability and resilience in their original habitats, facilitating at the same time the undertaken efforts regarding their conservation and potential domestication in artificial settings.

## 1. Introduction

Tulips are famous ornamental plants worldwide with high economic importance, therefore, the demand for new botanical tulips with interesting features is constantly high [1]. For example, the high demand for Greek botanical tulips has been satisfied to date by 24 nurseries located in four countries (Greece excepted), and the relatively high prices of the traded materials may showcase an online global market [1]. In addition, there are 41 botanic gardens around the world involved in the ex situ conservation of almost half of the Greek tulip species [1]. In Greece, all wild-growing tulips are protected by the Greek Presidential Decree 67/1981, occurring as wild-growing plants either in the Greek mainland or some of the Greek islands. These phytogenetic resources include 15 different species of the genus *Tulipa.* Among them, six are restricted to Greece (single-country endemics), while five are local sub-endemics in the Balkan or the Aegean regions extending to neighboring countries, and three are considered naturalized alien species originating from Asia [2]. Seven of these species are considered as threatened according to the criteria established by the International Union for Conservation of Nature (IUCN); two of them are assessed as critically endangered (CR), three as endangered (EN), and two as vulnerable (VU), all suffering mainly due to over-collection, habitat degradation and land use changes [1,3,4,5].

In terms of their specific habitat requirements, the Greek tulips have evolved various adaptations to their local environments in the wider spatial (phytogeographical) units of the country where they thrive. Likewise, distinct morphological and ecological species-specific characteristics have also been developed due to the above-mentioned adaptation process [1,2]. Such adaptation strategies are supported by the combined action of multiple elements (nutrients) which, once they are taken up by the plant root system, are involved in various biochemical reactions dictated by species-specific genotypes with individual variations [6]. Among the latter, macro-nutrients such as nitrogen (N), phosphorus (P), potassium (K), calcium (Ca), and magnesium (Mg) are considered necessary components for performing various metabolic processes, for protecting plants from abiotic and biotic stresses, or for developing their body structure [6,7,8]. Similarly, micro-nutrients such as iron (Fe), manganese (Mn), copper (Cu), zinc (Zn), and boron (B), or beneficial elements such as sodium (Na) are involved in various plant functions such as osmoregulation and electrochemical reactions [6,7,8].

As in any plant species, the particularities reflected in the nutrient status of Greek tulip species or the balance of essential macro- and micro-nutrients in their above-ground tissues or bulbs can provide valuable insights into their growth regimes, development patterns, and adaptation strategies to their local environments [9]. For example, it has been well established that different plant species thriving in similar or even identical soil environments may strongly differ in their uptake, transport, or accumulation strategies of nutrients due to the high specificity that each species develop in terms of its ionomic homeostasis [9]. Accordingly, the differences that might arise between several biotic or abiotic factors of different ecological environments may largely influence the ionome or the nutrient status between different species or those of different plant individuals belonging to the same species [7,10].

In this line, among several environmental conditions that exist in the immediate vicinity of any terrestrial growing plant (tulip plants as well), soil properties are considered critical factors since they play a crucial role in determining the availability of plant nutrients, and eventually in regulating their adaptation strategy and reproduction prerequisites dictated by each genotype [11]. Thus, differences that might be observed in properties such as soil texture, CaCO_3_ content, soil pH, electrical conductivity, or cation exchange capacity may reflect different patterns of nutrients accumulation or balance in different parts of the plants, since they generally play a critical role in shaping their overall ionome [12].

In addition to these abiotic factors, certain biotic factors such as microbes also impact the variations of elemental concentrations in plants thriving in their micro-environments [13,14,15]. Some of the most typical processes affected or triggered by soil microbial activity include the mineralization process of soil organic matter and the fixation of nitrogen through symbiotic microorganisms as well as the phosphorus or nitrogen acquisition by rhizosphere mycorrhizal fungi [8,16,17].

The domestication of wild plants with attractive characteristics is highly valued in the ornamental horticultural sector, especially when they possess rarity or endemism (uniqueness) [1,18]. Thus, a concerted effort to acquire specialized knowledge about the nutritional requirements of wild-growing Greek tulip species and the mycorrhizal fungi present in their rhizosphere could further improve their cultivation potential.

Since maintaining biodiversity is important for the overall health and sustainability of ecosystems [19], understanding the interaction of different species with biotic and abiotic factors in their environment is of crucial concern. To this end, nutrient cycling plays a major role in ecosystem functioning [20] and can act as an indicator of the health status of different ecosystems [21]. In this regard and considering that both natural and artificial landscapes are threatened by the ongoing and ever-increasing ecological crisis [19], it is essential to identify the properties of the habitats of different species for conservation management. Determining both biotic and abiotic conditions of various habitats can offer knowledge about the species’ needs in terms of nutrients including interrelationships thereof with other living factors such as arbuscular mycorrhizal fungi or microbes [19,21]. Thus, establishing species-specific profiles could possibly provide insight into distribution patterns followed regarding species and/or habitats of conservation concern.

This study is focused on the protected wild-growing tulips of Greece (some of which are also threatened with extinction) for which almost nothing is known about their natural nutrient status, thus no insight is currently available into their growth and adaptation to their natural environment or artificial settings. Therefore, it should be considered that constitutes a research gap that needs to be investigated.

Our initial hypothesis was that the status of essential macro- and micro-nutrients in wild-growing tulips of Greece could offer valuable insights into their growth, reproduction, and stress tolerance experienced in their natural habitats. Furthermore, we examined how this overall variance is affected by different functional groups of tulips and analyzed their distribution across different spatial (phytogeographical) units. Therefore, we aimed to identify patterns or groupings that could enhance our understanding of their adaptation strategies to their local environment or specific nutritional requirements for different tulip groups, while we consider that this information brings novelty to conservation management research.

The objectives of our study were (i) to explore the nutrient content profiles of wild-growing Greek tulips analyzing different plant parts (above-ground biomass, and subterranean bulbs); (ii) to investigate their interrelationships with respective soil properties (physicochemical and microbial) as proxies of ecological conditions under which these tulips naturally thrive in the wild; and (iii) to decipher the role that different phytogeographical units may play in shaping differences on the nutritional status of various Greek tulip species. Based on the above-mentioned, we aimed to shed light on Greek tulips’ adaptability and resilience in their original habitats, facilitating at the same time the undertaken efforts regarding their conservation and potential domestication in artificial settings.

## 2. Materials and Methods

### 2.1. Focal Greek Tulips

**Figure 1:**
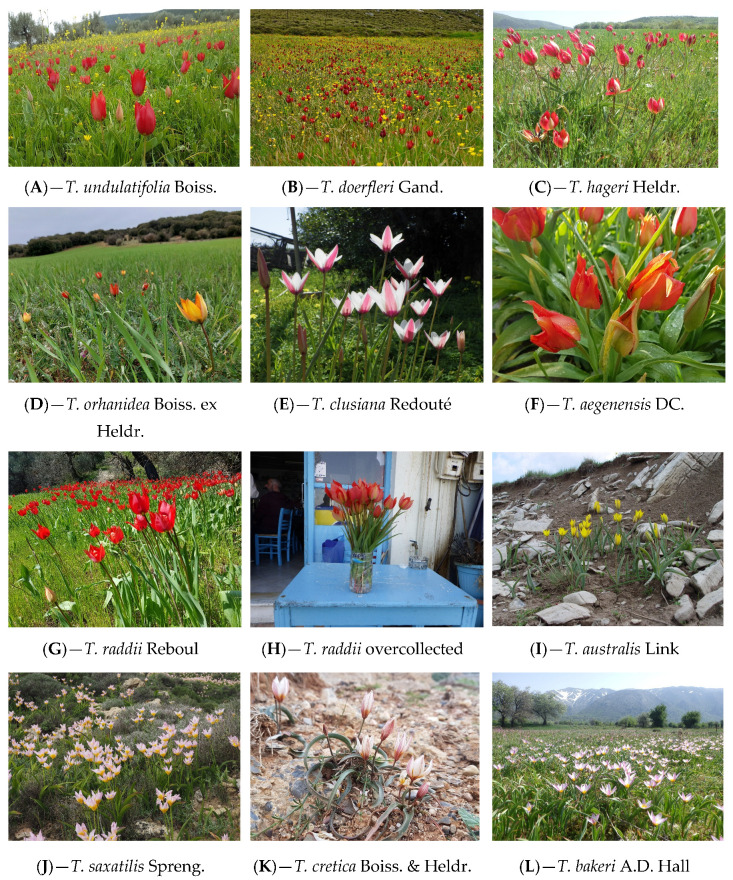

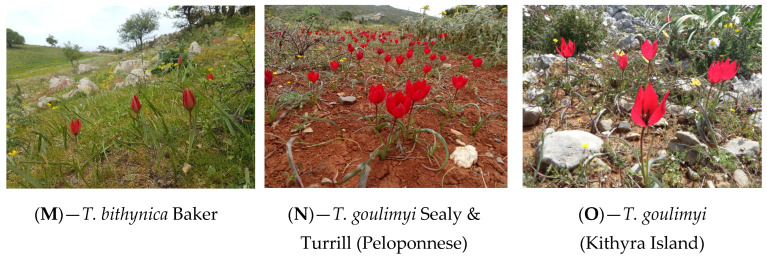
Flowering individuals of mainly segetal (**A**–**H**) and rock-dwelling or non-segetal (**I**–**O**) wild-growing tulips of Greece employing different biogeographical groups such as Greek endemic ones (*T. bakeri*, *T. cretica*, *T. doerfleri*, *T. goulimyi*, *T. hageri*, *T. orphanidea*), sub-endemic ones extending to Turkey and/or the Balkans (*T. bithynica*, *T. saxatilis*, *T. undulatifolia*), Mediterranean (*T. australis*) and Asiatic ones naturalized in Greece (*T. aegenensis*, *T. clusiana*, *T. raddii*).

**Figure 2:**
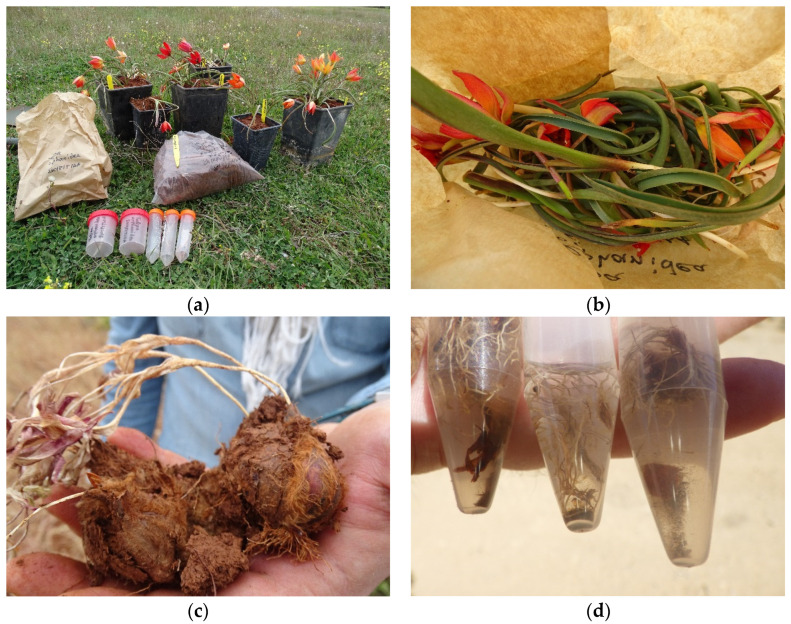
Examples of sampling materials from wild-growing tulip individuals of Greece collected with permission and studied herein: (**a**) propagation materials for ex situ conservation, soil and leaf samples for nutrient diagnostics, and root samples for mycorrhiza analysis collected from *Tulipa orphanidea*; (**b**) leaf samples of *Tulipa orphanidea*; (**c**) bulb samples of *Tulipa goulimyi*; and (**d**) Mycorrhiza samples from different individuals of *Tulipa bakeri*.

In this study, 13 (86.7%) of the Greek *Tulipa* species are included (Figure 1 and Figure 2, Table 1). Plant nomenclature of the Greek tulip species studied herein follow the Flora of Greece web version IV (https://portal.cybertaxonomy.org/flora-greece/intro, accessed on 22 February 2023).

### 2.2. Collection of Samplings

Samples from about 5–10 wild-growing individuals of each studied species were collected during botanical expedition in 2020 and 2021 in the frame of the research project TULIPS.GR (Figure 2, Table 1). The samples included specimens for ex situ conservation, soil samples, above-ground leaf samples, and below-ground bulbs for nutrient analysis as well as samples for mycorrhiza analysis (Figure 2). All collections were performed using a special permission issued yearly (182336/879 of 16 May 2019, 64886/2959 of 6 July 2020 and 26895/1527 of 21 April 2021) by the national competent authority, namely the Greek Ministry of Environment and Energy.

### 2.3. Classification of Studied Species into Functional Groups

The sampled wild-growing Greek tulip species were classified into different functional groups (Table 2) based on species-specific biological data [2], such as taxonomic identity and phytogeographical distribution, chorological origin, ecological preferences in terms of habitat types and altitudinal range and categories thereof, and threatened status according to IUCN (International Union for the Conservation of Nature) extinction risk assessments [3,4]. The following functional groups of Greek tulips were outlined:

1. Tulips of mainland Greece and insular tulips of Greece (North Aegean tulips and Cretan tulips);
2. Alien naturalized tulips and wild-growing Greek native tulips;
3. Greek endemic tulips, sub-endemic tulips, and tulips of wider distribution range;
4. Non-threatened and threatened tulips (critically endangered, endangered and vulnerable);
5. Tulips of small altitudinal range (lowland tulips, tulips of intermediate altitudes), tulips of wide altitudinal range (tulips of lowland to intermediate altitudes, tulips of intermediate to high altitudes) and tulips of very wide altitudinal range (tulips occurring from lowlands to high altitudes);
6. Tulips associated with agricultural habitat types, tulips associated with natural habitat types, and tulips occurring in both agricultural and natural habitat types;
7. Rock-dwelling tulips and tulips not occurring in rocky habitats;
8. Segetal tulips and tulips not occurring in agricultural habitats;
9. Early flowering tulips, mid-spring flowering tulips, and late flowering tulips.

### 2.4. Soil and Plant Analysis

A total of 34 surface soil samples (0–30 cm) were collected in the sampling area in which the respective plant material (tulip above-ground biomass, or bulb) was also collected. Soil samples were air-dried and passed through a 2-mm sieve. Then, they were analyzed in triplicate for the properties described hereafter. Determination of soil texture and respective distribution in sand, silt, and clay particles was conducted by the hydrometer method [22]. Organic carbon (C) was determined by the wet oxidation method [23] and CaCO_3_ was assessed using a calcimeter. The pH was measured in a 1:2 (*w/v*) water suspension, while the electrical conductivity of the soil solution was determined in the saturation extract (EC_se_). The sodium absorption ratio (SAR), representing an intensity factor of Na in the soil solution, relative to Ca and Mg, was calculated as follows:

(1) $$\mathrm{SAR}=\frac{{Na}^{+}}{\sqrt{\frac{{Ca}^{2+}+{Mg}^{2+}}{2}}}$$

where each chemical element symbol indicates a concentration in millimoles of charge per liter (mmol_c_ L^−1^) [24]. The hexamminecobalt(III) chloride ([Co(NH_3_)_6_]Cl_3_) method (ISO 23470) was used to assess the cation exchange capacity (CEC).

As far as the available macro- and micro-nutrients are concerned, soil available P was extracted using 0.5 M NaHCO_3_, pH 8.5, and was measured by the molybdenum blue-ascorbic acid method [25]. Extraction with 1 M KCl was used to determine both NO_3_-N and NH_4_-N, whereas the measurement was conducted with UV-Vis spectrometry and the sodium salicylate-sodium nitroprusside method, respectively [26]. Exchangeable cations (K, Ca, Mg, Na) were extracted with 1 M ammonium acetate (CH_3_COONH_4_), pH 7 [27]; K and Na were measured with flame photometry, while Ca and Mg were measured by atomic absorption spectrometry. The DTPA method [28] was used for Cu, Zn, Fe, and Mn extraction, which were measured by atomic absorption spectrometry as well. Boron was extracted with hot water and the determination was carried out with the azomethine-H method by UV-Vis spectrometry [29].

Sub-samples of the above-ground biomass or bulbs of each tulip species collected at the flowering stage were ashed at 500 °C for a four-hour minimum [30]. The ash was then dissolved in 2 M HCl following filtration, while the filtrate was used for the determination of P, K, Ca, Mg, Cu, Zn, Fe, Mn, and B employing the analytical methods described previously for soil analysis. In addition, the above-ground biomass and bulbs were analyzed for total N by the Kjeldahl method [31]. All plant samples were also analyzed in triplicate and their values are presented as their mean.

The wet sieving and decanting method with density gradient centrifugation described by [32] was used for arbuscular mycorrhizal fungi (AMF) spore extraction from 50 g soil samples. The spores were then counted and observed under a dissecting scope at 20–35× magnification and were grouped into morphotypes. 

### 2.5. Statistical Analysis

For each plant or soil parameter determined among different species and sampling sites, descriptive statistics were applied, while the three spatial (phytogeographical) regions of Greece (North Aegean Islands, Crete Island, and mainland Greece) were set as a factor with three levels, and analysis of variance (ANOVA) was conducted. The protected least significant difference (LSD) test was used for mean comparisons at *p* ≤ 0.05 to investigate differences between plant or soil parameters whereas correlation analysis and principal component analysis (PCA) was also applied to tulips’ nutrients content variables. In addition, linear or linearized single and multiple regression models were fitted to investigate the effects of soil properties on Greek tulips’ above-ground macro- and micro-nutrients content variability. All analyses were conducted using the Statgraphics software (STATGRAPHICS, CENTURION 18, version 18.1.12, STATPOINT TECHNOLOGIES, Inc., The Plains, VA, USA), and the PCA results were visualized using R software (V.4.2.2) with “ggplot2” package.

## 3. Results

### 3.1. Soil Properties of the Studied Samples

The descriptive statistics of the selected physicochemical properties of the soils are presented in Table 3. The coefficient of variation expressed as a percentage (CV, the ratio of the standard deviation to mean) for the general properties (Table 3) was in the range of 11.2–170.4%, indicating that for variables such as CaCO_3_, OC, EC_se_, total N, or clay content and CEC, there was a greater dispersion.

As far as soils’ fertility status is concerned, NO_3_-N, P, and K were among the soil available macro-nutrients with the largest variability, whereas the same was also the case with the micro-nutrients Zn, Fe, and Mn. On the contrary, concentration values of soil available Ca, NH_4_-N, as well as Cu and B turned out to be more stable with lower variations.

### 3.2. Nutritional Status of the Studied Greek Tulip Species

**Figure 3:**
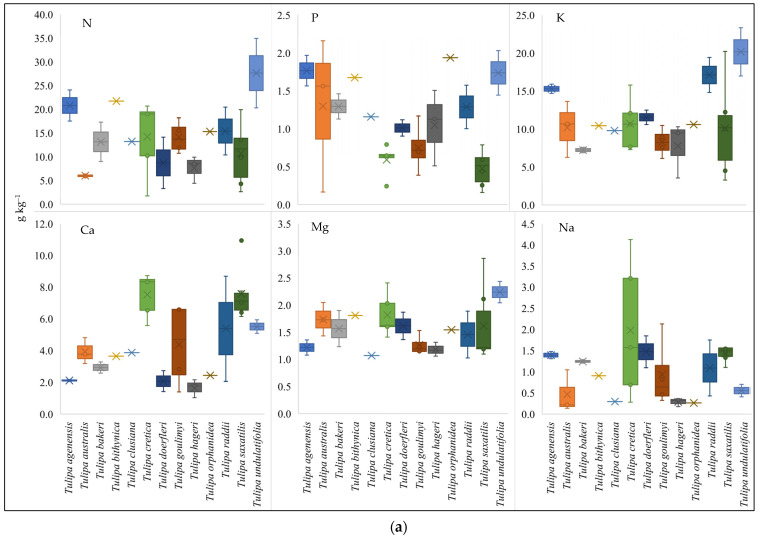

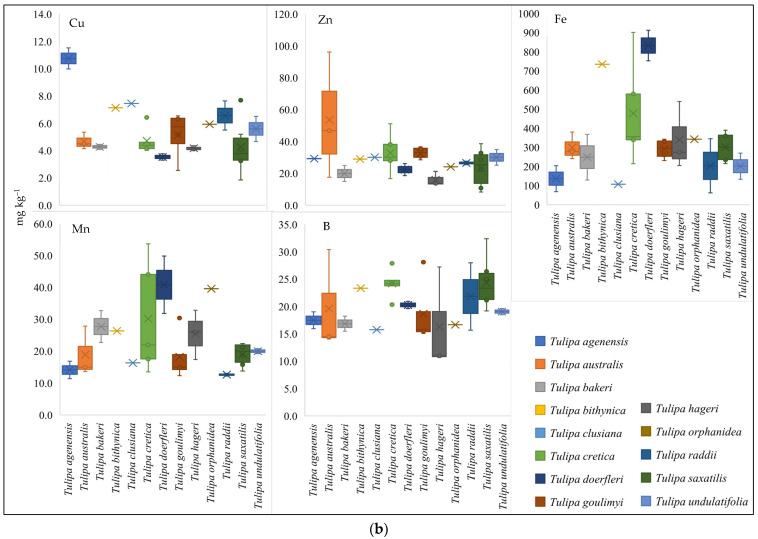
Boxplots showing concentrations of (**a**) macro-nutrients (N, P, K, Ca, and Mg) and beneficial element Na, and (**b**) micro-nutrients (Cu, Zn, Fe, Mn, and B) in above-ground biomass of wild-growing Greek tulip species. Box colors represent different *Tulipa* species.

The nutritional status of the above-ground biomass of the sampled Greek tulips was examined and the results are presented as boxplots in Figure 3, while the respective procedure was also followed for bulb samples (Supplementary Material Figure S1). As regards the above-ground biomass samples, the concentrations of macro- and micro-nutrients generally varied among species, while *T. undulatifolia* recorded the highest values for N, K, and Mg, *T. orphanidea* for P, whereas *T. saxatilis* and *T. cretica* for Ca, and N, accordingly. On the contrary, for the same elements (with the sequence of N, P, K, Ca, Mg, and Na, respectively), the lowest values were recorded for *T. australis*, *T. saxatilis*, *T. bakeri*, *T. hageri*, *T. clusiana,* and *T. orphanidea*.

Regarding the cationic micro-nutrient concentration, *T. doerfleri* was found to contain the highest values in Fe and Mn, while the same species recorded the lowest values in Cu. *T. australis* on the other hand, was found high in Zn above-ground biomass concentration while *T. agenensis* in Cu concentration. The lowest values in Zn, Fe, and Mn were recorded in *T. hageri*, *T. clusana*, *and T. raddii*, respectively. However, B concentration of above-ground biomass among species did not follow large variation patterns, and the distribution of its variance proved to be the most stable of all micro-nutrients studied.

### 3.3. Interrelationships between Tulips’ Essential Macro- and Micro-Nutrients Content

Pearson correlations between the set of nutrient content variables examined in the above-ground biomass of tulips revealed significant positive or negative correlations in each case and the results are shown in Table 4. Among them, worth noting are the positive correlations between the three major macro-nutrients (N, P, and K), while the same also applied as expected for Ca and Mg (*r =* 0.49, *p ≤* 0.01). In addition, B was found to be positively correlated with Ca, Mg, and Na (with *r* value ranging up to 0.65, *p ≤* 0.001), whereas negative correlations on contrary were observed between B and P (*r* = −0.65, *p ≤* 0.001). The latter was also negatively correlated with Ca and Na, while as far as the micro-nutrients are concerned, strong and positive correlations were also found between Fe and Mn (*r* = 0.62, *p ≤* 0.001).

### 3.4. Relationships between Rhizosphere’s Arbuscular Mycorrhizal Fungi (AMF) Spore Morphotypes, Soil Parameters, and Tulips Nutrients Content

The results from Pearson correlations concerning the arbuscular mycorrhizal fungi (AMF) spore morphotypes showed that among all studied nutrients of tulips’ above-ground biomass, they were significantly and positively correlated only with N (*r* = 0.4, *p* ≤ 0.05). As regards the soil parameters, on the contrary, a negative correlation was found with soil available NH_4_-N (*r* = −0.38, *p* ≤ 0.05), while the same was also the case with soil Mn and Fe extracted with DTPA, respectively (*r* = −0.36, *p* ≤ 0.05, *r* = −0.44, *p* ≤ 0.01). A negative correlation was found with regard to the soil texture parameter between AMF spore morphotypes and the percentage of sand (*r* = −0.34, *p* ≤ 0.05).

### 3.5. Nutrients Variability of Wild-Growing Greek Tulips and Functional Types Distribution

**Figure 4:**
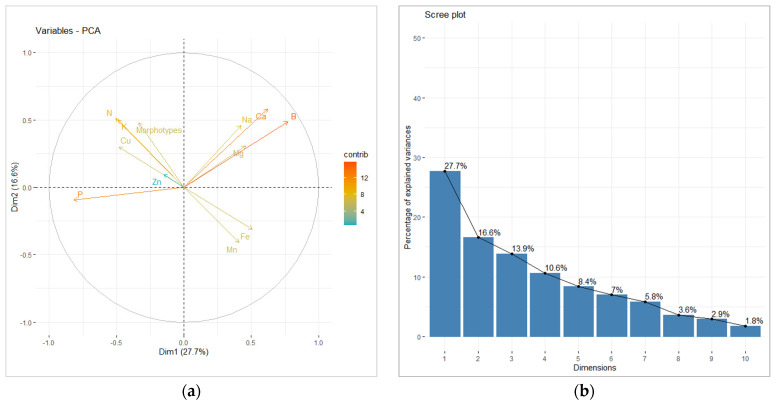
Principal component analysis (PCA) of (**a**) the studied macro- and micro-nutrients of tulips’ above-ground biomass (vectors), and (**b**) the respective contribution of each component to their total variability.

The PCA analysis (Figure 4a) revealed that the major portion of the total variance (almost 80%) of the studied variables (nutrients’ content of above-ground biomass, and fungi morphotypes) was grouped between five components, and the two of them explained 44.3% of it (Figure 4b). The variables that most contributed in the first two PCA axes were the elements B (15.7%) and Ca (13.6%), P (12.7%), N (9.8%), and K (9.2%), whereas Mn and Fe contributed mostly on the third component (30% and 26.7%, respectively).

**Figure 5:**
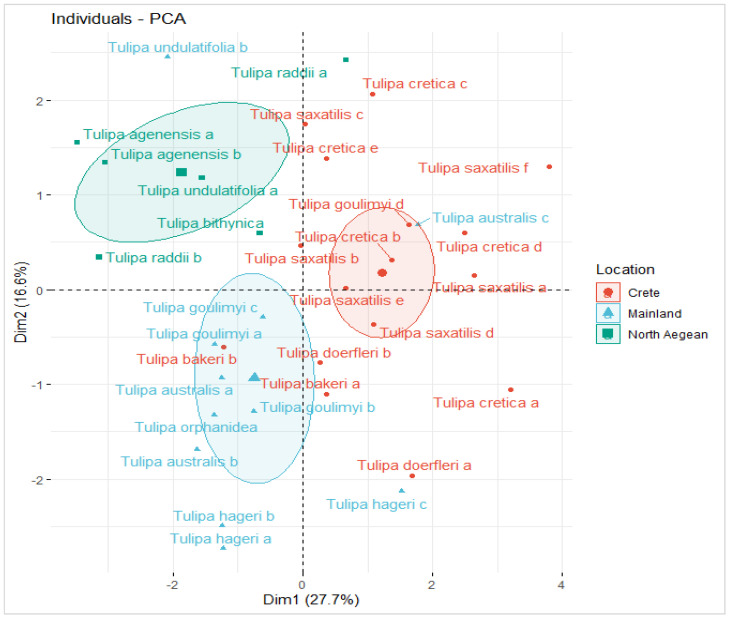
Distribution of each sampled tulip species as scattered among the first two components of the PCA (95% confidence ellipses), and respective grouping in terms of their specific spatial (phytogeographical) units (location) namely North Aegean Islands, Crete Island, and mainland Greece.

The distribution of individuals of the PCA analysis (sampled tulips species) as scattered between the first two components was further evaluated using the sampling location as categorial factor as well as the tulips’ functional properties to explore potential distribution similarity patterns. The results presented in Figure 5 reveal that between the three spatial (phytogeographical) units examined (North Aegean Islands, Crete Island, mainland Greece), the total nutrients’ variability produced a clear distinction among sampled species. More specifically, the samples of the North Aegean Islands were scattered in the upper left quadrant of the PCA plot (Figure 5), indicating a strong association with the respective variables of N, K, and Cu, and with the AMF spores morphotypes (Figure 4a). On the contrary, most of the Cretan samples were scattered in the upper right quadrant (Figure 5), showing respective association with B, Ca, Na, and Mg (Figure 4a). A corresponding association was also observed between the Cretan samples scattered in the lower wright quadrant with Fe and Mn variables, respectively.

**Figure 6:**
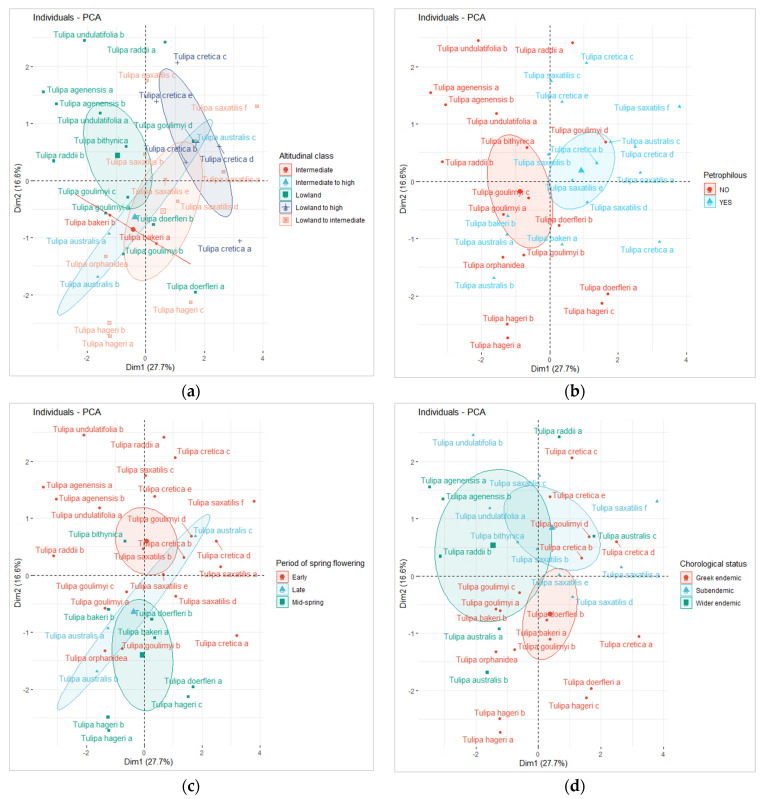
Distribution of each sampled tulip species as scattered betweem the first two components of the PCA (95% confidence ellipses), and respective grouping in terms of (**a**) different altitudinal classes; (**b**) rock-dwelling habit or not (petrphilous); (**c**) period of spring flowering; and (**d**) chorological status.

Respective though much less distinct patterns were also observed regarding the other functional groups of the studied tulip species outlined in Section 2.3 (Figure 6). For example, the rock-dwelling Greek tulips were mostly scattered in the positive side of the first component axis whereas all other tulip species occupied the negative side. Similarly, the mid-spring flowering Greek tulips were polarized in the most negative side of the second component axis compared with the early or late flowering ones. Concerning the altitudinal class, the lowland Greek tulip species primarily represented by the samples of the North Aegean Islands were scattered in the upper left quadrant of the PCA plot. The latter indicated a strong association of this group of tulips with the respective variables of N, K, and Cu, and with the AMF spores morphotypes as well. The distribution pattern of the chorological status was rather indistinct, denoting that the variability of the tulips’ nutrients should not be considered a major factor driving differences between these groups.

### 3.6. Effects of Soil Properties on Greek Tulips’ Above-Ground Macro- and Micro-Nutrients Content Variability

Linear or linearized single and multiple regression models were fitted among the dependent variables of tulips’ above-ground biomass elements concentrations, setting as independent variables the soil physicochemical properties. The results presented in Table 5 revealed significant relationships for all studied macro- and micro-nutrients, while parameters such as EC_se_ and CEC in some cases managed to explain almost up to 70% of nutrient’s concentration total variance (dependent variable, leaf P).

Except for the CEC and EC_se_ parameters, pH, CaCO_3_, clay, and OC content were also among the soil properties that contributed the most, while notably, respective soil available concentrations played a significant role in explaining their variance only in the case of Na, Cu, and Zn plants’ concentrations.

### 3.7. Evaluating Differences in Nutrients Profile and in Soil Properties between Different Spatial Units

The classification of tulip species in the three spatial (phytogeographical) units of North Aegean Islands, Crete Island and mainland Greece was set as a factor in the analysis of variance (ANOVA) that was conducted. The latter was chosen because of the clear grouping patterns that the PCA analysis provided (Figure 5) in conjunction with the relationships that were shown between tulips’ above-ground biomass elements concentrations and the soil physicochemical properties. Thus, potential statistical differences were investigated for three sets of variables: (a) macro- and micro-nutrient content of the tulips’ above-ground biomass alongside with the assessed number of fungal morphotypes found in each sample, (b) macro- and micro-nutrient content of bulbs, and (c) soil physicochemical properties.

The results presented in Table 6 showed that regarding the nutrient content of the above-ground biomass of Greek tulip species, statistically significant differences were found for most of the cases (except Mg, and Zn concentration mean values). More specifically, North Aegean tulips were associated with the higher concentrations in the three of the major macro-nutrients (N, P, K) alongside Cu as compared with the respective elements measured in tulips sampled from Crete. The same was also the case with the fungal morphotypes while North Aegean tulips presented the lowest mean values in Ca, Na, Fe, and Mn compared to the Cretan ones. On the contrary, the mean values of the above-mentioned variables recorded in the tulips from mainland Greece were either polarized with the respective values of North Aegean tulips (P, Ca, and Na) or with those in tulips from Crete (N, K, Cu, and fungi morphotypes) or they were clustered in the middle without differing significantly with none of them (Fe, and Mn).

Interestingly, agreement with the above results was only noted for exchangeable K and Cu extracted with DTPA in terms of available macro- and micro-nutrients in soil (Table 7), while even contradictory results were recorded as in the case of soil available Ca. More particularly, even though its concentration in soils sampled from the North Aegean Islands was found high (as exchangeable Ca), the corresponding values of the above-ground biomass were significantly lower compared with those of Crete. In addition, it is noteworthy that unlike the above-ground biomass, nutrient content in tulip bulbs did not show corresponding differences between location groups linked with sample collections (data not shown). On the other hand, among several soil properties studied (Table 7), CEC values were found to be significantly higher in North Aegean samples as compared with those from Crete or mainland Greece, whereas this trend also stood for the clay content parameter, in which, however, significant differences occurred only between North Aegean Islands and Crete. In addition, the mean values of CaCO_3_ content differed significantly between the North Aegean Islands and the mainland (with higher values in the North Aegean).

## 4. Discussion

Our study aimed to provide for the first time a comprehensive overview of the wild-growing Greek tulip species focusing on their nutritional profiles and their interrelationships with the specific soil conditions that prevail in the small environmental scale of their original habitats.

As seen from the respective results concerning the soil properties of the studied samples, large variance patterns were observed among several soil parameters such as CaCO_3_, OC%, EC_se_, and total N, or clay content and CEC. The latter indicates that the Greek tulip species are wild-growing plants in habitats characterized by diverse soil conditions, while this natural spatial variability of soil properties might adversely affect the soil availability of nutrients required for plant growth. For instance, it is well established that the use of CEC parameter as a covariate significantly improves prediction models of K availability in soils [33,34], whereas EC_Se_ has also been suggested that can reduce the experimental error variance and thus to increase the precision of estimates of available soil P [35,36].

Similar impacts on the availability of soil nutrients derived by respective soil parameters could also be reflected in the tulips’ nutrients content profile (Figure 3) to the extent that their variability is explained by such impacts. Previous studies investigating leaf ionomic responses of different plant species grown to diverse edaphic sites [9] have found that the influence of soil parameters to the respective element variations in plants were strong but limited within the phylogenetic preset; however, for some cases such as leaf P concentration, for example, soil condition played a more important role compared to phylogenetic factors. Similar conclusions have also been reached by other researchers suggesting that ionomic variations are mainly driven by environmental conditions [37,38]. Based on our results, we consider that the above assumptions are more evident herein, given that we studied different members (species) of the same genus in which phylogenetic variance is strongly reduced. More specifically, our study showed that significant relationships occurred (from 21.6% up to 67.1%) between the above-ground concentrations of all investigated macro- and micro-nutrients and the respective soil parameters. In addition, corroborating with previous findings [9], tulips P concentration did appear to be strongly and significantly affected by soil parameters, with the EC_se_ playing key role in this effect.

Some of the specific correlation patterns among nutrients content of above-ground biomass (Table 4) have also been reported by other researchers, although such trends could be attributed to several factors. For instance, previous studies have reported that the common source for the element pairs could explain positive correlations among elements in the plants’ biomass [39]. The above assumption could stand in the case of the observed Fe–Mn positive correlations detected herein given the fact that these elements were also significantly influenced by specific soil properties such as pH and OC (Table 5).

Hence, similar solubility rates driven from respective soil pH variations, as well as similar decomposition rates of soil organic matter releasing proportionally equal amounts of the respective micro-nutrients probably bound in soils’ organic matter could provide a possible explanation mechanism for their positive correlations in tulips’ plant tissue content. In addition, the tendency of elements with similar physicochemical properties to share or compete for pathways or transport systems accumulating them in leaves [9,40] might partially explain the observed negative B-P correlations in this study, or the positive Ca–Mg interactions. In this line, the positive correlations between B and Ca could be also attributed to the fact that B tends to keep Ca in a soluble form within the plant [41].

The correlation results regarding the AMF spore morphotypes and their relationships with soil or plant nutrients parameters are also of interest. It is well known that the AMF symbiosis is important for plant P nutrition, and that higher P levels suppress the symbiosis [42]. It is also known that AMF may also contribute to plant N nutrition [43,44], and the positive correlation between AMF spore morphotypes with tulip tissue N points to that direction. On the other hand, previous studies have shown that elevated soil N may reduce AMF spore richness and abundance [45], which is in line with the negative correlation detected in this study between AMF spore morphotypes and the soil available NH_4_-N. However, N effects may also be related to N form and plant species [46]. Based on the tissue and soil N correlation, we would expect N fertilization to reduce AMF spore morphotypes and the importance of AMF in tissue N.

The micro-nutrients Fe and Mn are important in many physiological roles in plants, and previous studies have found that N addition may increase foliar Mn suppressing photosynthetic rates [47]. On the other hand, Fe and Mn may function as soil redox indicators, being more mobile at reduced soil conditions, which could also be detrimental for AMF and may offer explanation for the negative correlation of AMF spore morphotypes with these two micro-nutrients in this study. Soil texture is another important soil parameter for AMF spores, with clay rather than sand favoring their presence [48]. Other factors such as climate or elevation that were found to be important for AMF spores [49] exceeded the purposes of the present study and thus they were not examined.

The PCA results in conjunction with the respective ANOVA findings indicated that the distinction observed among sampled tulip species between the three spatial (phytogeographical) units examined (North Aegean Islands, Crete Island, and mainland Greece) might be ascribed to a large extent to differences found in specific soil properties. However, our results indicate that the detected variance in all variables studied regarding mainland Greece was expectedly high due to the comparatively large geographic scale and concomitant spatial heterogeneity of the latter spatial unit including seven phytogeographical regions. Thus, to avoid scale-dependent biases, the discussion made hereafter has been limited to comparisons between Crete and the North Aegean Islands since both spatial units are insular and of comparable size and are referred to as specific phytogeographical regions in the context of the Greek flora (see https://portal.cybertaxonomy.org/flora-greece/annotations, accessed 25 March 2023). In this way, the samples of tulip species from the North Aegean Islands clustered in the upper left side of the PCA plot were related to high levels of nutrients such as N, K, or Cu, a fact which was also documented by the ANOVA results that showed statistically significant higher values of these nutrients in tulip species of this phytogeographical unit. In the same line, the above was also the case for some elements such as Ca, Mg, Na, Fe, and Mn originated from tulips sampled from Crete Island (Table 6).

Considering that specific soil properties significantly influenced the variability of tulips nutrient content (Table 7), the respective ANOVA results concerning differences in soil properties among the studied spatial (phytogeographical) units may provide some interpretation of the above variance. For example, the soil samples of the phytogeographical unit of the North Aegean Islands are characterized by high values in CEC and clay content, respectively, a fact also reflected in the corresponding amounts of exchangeable soil K which were found to be correspondingly high as well.

We consider that the above trend is reflected in the significantly higher amounts of leaf K found in the North Aegean tulip samples whereas the same could also be the case with the correspondence that appeared between the significantly higher levels of foliar Cu and soil available Cu, respectively. The above remarks could fall into a category of cases in which a direct relation between soil available nutrients and their respective concentration in plant tissues occurs, as this has also been reported by several correlation studies and agricultural field experiments [50].

Nevertheless, the above rationale cannot explain the case of the significantly higher concentrations in N or P found in tulips sampled in the North Aegean Islands as compared with Crete, for example, since the physicochemical soil properties did not seem to have any effect in these variations. This leaves open the possibility that tulip species of the North Aegean Islands may be more efficient in P or N uptake, and/or they develop more efficient symbiosis with AMF. The higher number of the AMF spore morphotypes found in the samples of the North Aegean Islands indicated that the latter may be the case [51].

In the same line, the contradictory results that were recorded between the higher tulips’ plant Ca content in samples of the North Aegean Islands compared with those from Crete for example (although with non-recorded significant differences in soil available Ca) could not be explained based on the direct soil-plant connection concept, as previously offered for leaf K. Instead, species factors and respective adaptation mechanisms developed to respond to soil environmental specificities might provide some explanation. For instance, it is well established in the literature that when the presence of K is dominant in soil and in plants, antagonistic relationships may occur between other basic cations like Mg or Ca [52,53], a fact which might be true in our case, considering the significantly higher amounts of soil and plant K detected in the North Aegean samples.

Concerning the role that the functional groups of tulips outlined herein might play into potential differences detected on their nutrients content profile, the results presented in Figure 6 did not provide as clear information as the grouping between spatial (phytogeographical) units did. Nevertheless, the combined reading of the overall results of the present work can significantly contribute to a more comprehensive insight into the mechanisms of adaptation of the specific tulip species to their local environments. For example, PCA findings referring to the samples of the North Aegean may be interpreted as follows. These particularly lowland, non-petrophilous (rock-dwelling) and early flowering species (Figure 6), which naturally thrive in clayey soils with high levels of CEC showed higher number of the AMF spore morphotypes, while they were also associated with high levels of nutrients such as N, K, and Cu (Figure 4a). Thus, we consider that the above-mentioned combined information is crucial not only for providing conservation management insights of wild-growing tulips, but also for contributing on future domestication efforts as well.

Overall, the functioning of ecosystems is critical for the maintenance of biodiversity, the provision of ecosystem services, and the sustainability of human societies [19]. The current study has shed light on the nutrient acquisition patterns of 13 wild-growing Greek tulips originating from different phytogeographical units of this small but extremely diverse country in relation to soil properties and AMF morphotypes. Given that natural ecosystems are multifaceted and dynamic [54], these results are important for future conservation efforts and possible sustainable utilization plans concerning the wild-growing Greek tulip species and their original habitats. All the data furnished herein can be further exploited in attempts to cultivate ex situ in a sustainable way the diversity of the wild-growing Greek tulip species (*n* = 15) and finally domesticate them in artificial settings. Facilitating the latter is a complex and multi-dimensional process given that each of these wild-growing tulips originates from different environmental conditions, has different ecological preferences and has developed different natural adaptations [1,55]. To this end, the data generated herein can be used in the future to formulate species-specific fertilization guidelines to be followed in artificial settings which may benefit to a large extent from the insight into the species-specific nutritional needs, elemental uptake potential, and transport as detected in the original wild habitats of the wild-growing Greek tulips studied herein.

## 5. Conclusions

Despite the economic interest in tulip hybrids and botanical tulips worldwide [1], no insight is practically available to date into the protected wild-growing tulips of Greece, especially in terms of their growth and adaptation to their natural environment or artificial settings. Based on the latter research gap, our study showed that soil variables played a significant role in shaping wild-growing Greek tulips’ nutrient content, explaining up to 67% of the detected variability as in the case of the above-ground plant tissue P. In addition, respective correlation patterns were found between tulips’ essential nutrients. Among them, the observed Fe–Mn positive correlations could be attributed to similar solubility rates from respective soil pH variations, while the observed negative B–P correlations or the positive Ca–Mg interactions could be attributed to the tendency of elements with similar physicochemical properties to share or compete for common pathways or transport systems within the plants. The PCA revealed that between the three spatial (phytogeographical) units examined (North Aegean Islands, Crete Island, and mainland Greece), the total variability of tulips’ nutrient content produced a clear distinction among sampled species. In addition, the samples of the North Aegean Island were associated with nutrients such as N, K, and Cu, and with high numbers of AMF morphotypes, whereas the samples of the Crete Island were associated with nutrients such as B, Ca, Na, Mg, Fe, and Mn. This was further confirmed by the ANOVA results which showed corresponding statistically significant differences in both the tulips’ nutrient content and the studied soil properties as well. Among them, the significantly higher amounts of leaf K found in the North Aegean tulip samples could be attributed to the high values in CEC and clay content, respectively, a fact also reflected in the corresponding high amounts of exchangeable soil K. In addition, the significantly higher concentrations in N or P found in tulips sampled in the North Aegean Islands might be ascribed to a more efficient AMF symbiosis of the corresponding species, as has been indicated by the higher number of the AMF spore morphotypes found in the North Aegean samples. Therefore, our study sheds light on Greek tulips’ adaptability and resilience in their original habitats, facilitating at the same time the undertaken efforts regarding their potential domestication in artificial settings.

## Figures and Tables

**Table 1:** Collection details and IPEN (International Plant Exchange Network) accession numbers associated with the living material samples regarding the *Tulipa* spp. from Greece studied herein. Different *Tulipa* spp. are arranged alphabetically. For each species, the letter in the parentheses denotes different tulip samples collected (1–6 samples per species).

*Tulipa* Species	IPEN Accession	Altitude (m)	Phytogeographical Unit of Greece	Latitude (North)	Longitude (East)
*T. agenensis* (a)	GR-BBGK-1-22,2	101	North Aegean Islands	38.33376	26.081543
*T. agenensis* (b)	GR-BBGK-1-22,15	99	North Aegean Islands	38.333713	26.081833
*T. australis* (a)	GR-BBGK-1-21,115	1554	Mainland	40.096552	21.114582
*T. australis* (b)	GR-BBGK-1-21,116	1856	Mainland	40.096552	21.114582
*T. australis* (c)	GR-BBGK-1-22,54	477	Mainland	37.809379	23.941191
*T. bakeri* (a)	GR-BBGK-1-21,8	1057	Crete	35.330758	23.896977
*T. bakeri* (b)	GR-BBGK-1-22,28	1065	Crete	35.331772	23.907389
*T. bithynica* (a)	GR-BBGK-1-22,19	870	North Aegean Islands	39.161205	26.066789
*T. clusiana* (a)	GR-BBGK-1-22,1	422	North Aegean Islands	38.304548	26.056388
*T. cretica* (a)	GR-BBGK-1-21,79	12	Crete	34.957198	25.099697
*T. cretica* (b)	GR-BBGK-1-21,83	57	Crete	35.549880	24.150415
*T. cretica* (c)	GR-BBGK-1- 22,7	131	Crete	35.551054	24.147271
*T. cretica* (d)	GR-BBGK-1-22,9	158	Crete	35.222128	26.212745
*T. cretica* (e)	GR-BBGK-1-22,21	974	Crete	34.957373	25.100580
*T. doerfleri* (a)	GR-BBGK-1-21,106	743	Crete	35.207935	24.560692
*T. doerfleri* (b)	GR-BBGK-1-22,33	773	Crete	35.213769	24.567137
*T. goulimyi* (a)	GR-BBGK-1-21,39	415	Mainland *	36.16800	22.96600
*T. goulimyi* (b)	GR-BBGK-1-21,4	411	Mainland *	36.2531000	22.9677000
*T. goulimyi* (c)	GR-BBGK-1-21,41	616	Mainland	36.8283000	22.9475000
*T. goulimyi* (d)	GR-BBGK-1-22,25	177	Crete	35.503031	23.754020
*T. hageri* (a)	GR-BBGK-1-21,67	516	Mainland	40.799011	23.389939
*T. hageri* (b)	GR-BBGK-1-21,102	428	Mainland	40.421873	23.694688
*T. hageri* (c)	GR-BBGK-1-22,55	544	Mainland	38.190514	23.794406
*T. orphanidea*	GR-BBGK-1-21,44	876	Mainland	37.30900	22.42200
*T. raddii* (a)	GR-BBGK-1-21,285	183	North Aegean Islands	38.28275	26.07461
*T. raddii* (b)	GR-BBGK-1-22,3	381	North Aegean Islands	38.319157	26.055302
*T. saxatilis* (a)	GR-BBGK-1-21,105	721	Crete	35.213210	24.564025
*T. saxatilis* (b)	GR-BBGK-1-22,8	518	Crete	35.419784	23.740193
*T. saxatilis* (c)	GR-BBGK-1-22,14	95	Crete	35.074496	25.806127
*T, saxatilis* (d)	GR-BBGK-1-22,2	599	Crete	35.176982	24.997673
*T. saxatilis* (e)	GR-BBGK-1-22,27	531	Crete	35.419784	23.740193
*T. saxatilis* (f)	GR-BBGK-1-22,43	830	Crete	35.1696440	25.4882520
*T. undulatifolia* (a)	GR-BBGK-1-22,4	25	North Aegean Islands	38.203085	26.030258
*T. undulatifolia* (b)	GR-BBGK-1-22,11	445	Mainland	38.090000	23.230000

* Kythira island is situated between mainland Greece and Crete but here is classified into mainland Greece due to stronger floristic similarities.

**Table 2:** Overview of the functional groups of the sampled wild-growing Greek tulip species (*Ν* = 13) based on biological (taxonomical, phytogeographical, chorological, and ecological) data [2] and threatened status according to IUCN (International Union for the Conservation of Nature) extinction risk assessments [3,4]: CR—critically endangered; EN—endangered; VU—vulnerable.

Tulips (*Tulipa* spp.)	Phytogeographical Status	Chorological Status	Threatened Status	Altitudinal Class (Altitudinal Range in m)	Habitat Types	Rock-Dwelling	Segetal	Flowering
*T. agenensis*	Naturalized alien	Irano-Turanian (Chios Island, Greece)	No	Lowland (0–300)	Agricultural ^1^	No	Yes	Early
*T. australis*	Wild-growing native	Mediterranean-SW Asiatic	No	Intermediate to high (500–2000)	Natural ^2^	Yes	No	Late
*T. bakeri*	Wild-growing native	Greek endemic (Crete)	Yes (CR)	Intermediate (700–1300)	Agricultural and natural ^3^	Yes	No	Mid-spring
*T. bithynica*	Wild-growing native	Subendemic (Lesvos, Greece-Anatolia)	No	Lowland (200–800)	Agricultural and natural ^4^	No	No	Mid-spring
*T. clusiana*	Naturalized alien	Irano-Turanian (Chios Island, Greece)	No	Lowland (100–600)	Agricultural ^5^	No	No	Early
*T. cretica*	Wild-growing native	Greek endemic (Crete)	Yes (EN)	Lowland to high (0–2100)	Natural ^6^	Yes	No	Early
*T. doerfleri*	Wild-growing native	Greek endemic (Crete)	Yes (CR)	Lowland (400–800)	Agricultural ^7^	No	Yes	Mid-spring
*T. goulimyi*	Wild-growing native	Greek endemic (Peloponnese, nearby islands to Crete)	Yes (VU)	Lowland (0–900)	Natural ^8^	No	No	Early
*T. hageri*	Wild-growing native	Greek endemic (Sterea Hellas, Peloponnese and north Greece)	Yes (EN)	Lowland to intemediate (100–1200)	Agricultural and natural ^9^	No	Yes	Mid-spring
*T. orphanidea*	Wild-growing native	Greek endemic (Sterea Hellas, Peloponnese)	Yes (EN)	Lowland to intemediate (700–1600)	Agricultural and natural ^10^	No	Yes	Early
*T. raddii*	Naturalized alien	East Mediterranean (Chios island)	No	Lowland (0–400)	Agricultural ^11^	No	Yes	Early
*T. saxatilis*	Wild-growing native	Subendemic (South Aegean, Anatolia)	No	Lowland to intemediate (200–1300)	Agricultural and natural ^12^	Yes	No	Early
*T. undulatifolia*	Wild-growing native	Subendemic (Balkan-Anatolia)	Yes (VU)	Lowland (100–800)	Agricultural ^13^ and natural	No	Yes	Early

^1^: Clay cereal fields and terraced olive groves. ^2^: Rocky flats, screes, meadows, open woodland, verges of mountain roads, and rocky slopes. ^3^: Mountain plateau field margins, in scrub or by gravelly stream sides. ^4^: Terraced olive groves and open *Castanea* woodland. ^5^: Seasonally wet sites with cultivated and fallow fields. ^6^: Open habitats and phrygana with rich terra rossa or rocky and stony mountain slopes. ^7^: Cultivated and fallow fields and olive groves. ^8^: Terra rossa with phrygana. ^9^: Xeric Mediterranean phrygana and grassland or in agricultural habitats. ^10^: Meadows and formerly cultivated land in dolines. ^11^: Deep clay terraced olive and mastic groves as well as in cereal fields. ^12^: Rock-dweller in rocky limestone hills and flats. ^13^: Soil pockets on rocky and stony slopes with phrygana or open woodland or as a weed in cultivated and fallow fields and olive groves.

**Table 3:** Descriptive statistics of the studied physicochemical soil properties and the respective soil available concentrations of macro- and micro-nutrients. SD: standard deviation; CV: coefficient of variation; OC: organic carbon; CEC: cation exchange capacity; EC_se_: electrical conductivity of the saturation extract; Kex, Naex, Caex, Mgex: exchangeable amounts of K, Na, Ca, Mg, extracted with the ammonium acetate method; and B-HW: Boron extracted with the hot water method.

Variable	Min.	Max.	Median	Mean	SD	CV%
Sand (%)	20.8	78.2	49.6	48.9	13.8	28.2%
Silt (%)	10.4	53.2	26.7	28.1	9.3	33.1%
Clay (%)	6.4	47.0	18.2	23.0	12.8	55.4%
pH	5.20	7.90	7.65	7.21	0.81	11.2%
CaCO_3_ (%)	0.0	62.4	2.1	9.0	15.4	170.4%
OC (%)	0.64	12.12	1.39	2.39	2.30	96.3%
Total N (%)	0.08	0.80	0.16	0.22	0.16	72.6%
C/N	6.50	15.30	9.60	9.60	2.00	20.8%
CEC (cmol_c_ kg^–1^)	7.2	56.9	24.4	24.3	12.5	51.7%
EC_se_ (ds m^−1^)	0.14	2.64	0.45	0.57	0.50	84.7%
Kex (mg kg^–1^)	34	900	258	275	214	77.8%
Naex (mg kg^–1^)	12.7	96.3	49.2	47.9	20.5	42.9%
Caex (mg kg^–1^)	616	8472	3856	3663	1981	54.1%
Mgex (mg kg^–1^)	97	884	225	357	256	71.6%
NO_3_-N (mg kg^–1^)	1.1	98.7	7.3	13.0	17.5	134.5%
NH_4_-N (mg kg^–1^)	2.6	48.3	11.8	12.5	8.2	65.1%
P-Olsen (mg kg^–1^)	0.90	45.20	4.45	6.16	7.95	129.0%
Cu-DTPA (mg kg^–1^)	0.64	2.77	1.26	1.42	0.60	39.4%
Zn-DTPA (mg kg^–1^)	0.31	7.09	0.71	1.33	1.50	114.4%
Fe-DTPA (mg kg^–1^)	3.6	95.0	20.5	27.7	23.7	85.7%
Mn-DTPA (mg kg^–1^)	5.4	115.2	20.0	27.4	21.7	79.0%
B-HW (mg kg^–1^)	0.25	1.81	0.56	0.67	0.40	53.6%

**Table 4:** Pearson correlation matrix with respective *r* values between above-ground biomass concentrations of the studied macro- and micro-nutrients of the wild-growing Greek tulip species. Asterisks (*, **, ***) indicate significant *r* values at *p* < 0.05, 0.01, and 0.001, respectively; *n* = 34.

	N	K	Na	Ca	Mg	Cu	Zn	Fe	Mn	P
K	0.31 ^a^									
Na	0.21	−0.04								
Ca	−0.17	0.09	0.38 *							
Mg	−0.22	−0.09	0.15	0.49 **						
Cu	0.35 *	0.11	−0.17	−0.14	0.10					
Zn	0.44 **	0.19	0.07	−0.01	−0.24	0.09				
Fe	−0.28	−0.36 *	0.10	0.14	0.39 *	−0.34	−0.10			
Mn	0.01	−0.31 ^a^	0.11	−0.10	0.17	−0.23	0.07	0.62 ***		
P	0.39 *	0.40 *	−0.40 *	−0.57 ***	−0.13	0.46 **	0.22	−0.27	0.02	
B	−0.19	−0.16	0.57 ***	0.65 ***	0.49	−0.20	−0.04	0.37 *	0.15	−0.65 ***

^a^ *p* = 0.08.

**Table 5:** Single and multiple regression models between the tulips’ above-ground biomass elements concentrations (Y) and the respective soil physicochemical properties (X). *r*^2^: coefficient of determination; OC: organic carbon; CEC: cation exchange capacity; EC_se_: electrical conductivity of the saturation extract; and Naex: exchangeable amounts of Na extracted with the ammonium acetate method; *n* = 34.

Dependent Variable (Y)	Independent Variable (X)	Model	*r^2^* %	*p*-Value
N	Clay	Simple linear	32.2	0.0005
P	EC_se_, CEC	Multiple reciprocal-Y	67.1	0.0000
K	CaCO3, OC%, CEC	Multiple linear	58.2	0.0000
Ca	pH, EC_se_	Multiple linear	49.6	0.0000
Mg	EC_se_	Double squared	43.1	0.0000
Na	Naex	Double reciprocal	56.4	0.0000
Cu	Cu-DTPA	Double squared	22.1	0.0050
Zn	Zn-DTPA, pH, EC_se_	Multiple linear	57.5	0.0000
Fe	pH, OC%	Multiple linear	21.6	0.0258
Mn	pH, OC%	Multiple linear	47.5	0.0001
B	EC_se_	Squared-Y square root-X	38.3	0.0001

**Table 6:** Mean values of macro- and micro-nutrients’ concentrations in above-ground biomass of the studied tulip species or fungi morphotypes in terms of their specific phytogeografical unit (Location) namely North Aegean Islands, Crete Island, and mainland Greece. SE: standard error; NS: non-significant. Within each element or arbuscular mycorrhizal fungi (AMF) spores morphotype, different letters indicate significant differences among means, employing the protected LSD test, at *p* ≤ 0.05.

Location	Mean	SE	Mean	SE	Mean	SE	Mean	SE	Mean	SE	Mean	SE
	Ν		Ρ		Κ		Ca		Mg		Na	
	(g kg^−1^)											
North Aegean Islands	18.3 a	(1.8)	1.49 a	(0.12)	15.4 a	(1.8)	3.94 b	(0.91)	1.47 a	(0.17)	0.93 b	(0.22)
Crete Island	12.0 b	(1.6)	0.68 b	(0.09)	10.0 b	(1.0)	6.27 a	(0.65)	1.65 a	(0.13)	1.63 a	(0.23)
Mainland Greece	10.3 b	(1.5)	1.24 a	(0.20)	9.4 b	(1.1)	3.27 b	(0.56)	1.52 a	(0.13)	0.43 b	(0.09)
*p* F-test	0.021		<0.001		0.011		0.007		NS		<0.001	
	Cu		Zn		Fe		Mn		B		AMF spore morphotypes	
	(mg kg^−1^)											
North Aegean Islands	7.74 a	(0.91)	27.9 a	(0.8)	154 b	(44)	16.6 b	(2.0)	19.5 ab	(1. 8)	4.50 a	(0.62)
Crete Island	4.24 b	(0.35)	26.5 a	(2.8)	414 a	(62)	27.1 a	(3.1)	23.1 a	(1.1)	2.75 b	(0.49)
Mainland Greece	5.23 b	(0.31)	33.0 a	(7.1)	316 ab	(28)	19.6 ab	(2.2)	17.3 b	(1.9)	2.18 b	(0.46)
*p* F-test	<0.001		NS		0.023		0.048		0.022		0.044	

**Table 7:** Mean values of macro- and micro-nutrients’ soil available concentrations and respective soil properties of the sampled soil sites in terms of the specific phytogeographical unit (location) namely North Aegean Islands, Crete Island, and mainland Greece. SE: standard error; NS: non-significant. Within each soil available element, or soil property, different letters indicate significant differences among means, employing the protected LSD test, at *p* ≤ 0.05.

**Location**	**Mean**	**SE**	**Mean**	**SE**	**Mean**	**SE**	**Mean**	**SE**	**Mean**	**SE**	**Mean**	**SE**	**Mean**	**SE**
	**Clay** **(%)**		**pH**		**CaCO_3_** **(%)**		**OC** **(%)**		**N-Total** **(%)**		**CEC** **(cmol_c_ kg^−1^)**		**EC_se_** **(ds m^−1^)**	
North Aegean Islands	31.9 a	(4.5)	7.51 a	(0.22)	21.6 a	(7.1)	1.86 a	(0.62)	0.18 a	(0.04)	35.2 a	(1.6)	0.61 a	(0.11)
Crete Island	17.1 b	(2.6)	7.29 a	(0.17)	8.78 ab	(4.03)	2.81 a	(0.73)	0.25 a	(0.05)	21.6 b	(3.6)	0.71 a	(0.15)
Mainland Greece	25.9 ab	(3.9)	6.90 a	(0.31)	1.40 b	(0.64)	2.10 a	(0.48)	0.21 a	(0.04)	21.1 b	(3.0)	0.33 a	(0.06)
*p* F-test	0.020		NS		0.020		NS		NS		0.029		NS	
**Location**	**Mean**	**SE**	**Mean**	**SE**	**Mean**	**SE**	**Mean**	**SE**	**Mean**	**SE**	**Mean**	**SE**		
	**NO_3_-N**		**NH_4_-N**		**P-Olsen**		**Κ-ex**		**Ca-ex**		**Mg-ex**			
	**(mg kg^−1^)**													
North Aegean Islands	13.6 a	(4.3)	7.81 b	(1.14)	6.10 a	(2.55)	473 a	(114)	5103 a	(222)	627 a	(104)		
Crete Island	15.4 a	(5.9)	10.9 ab	(1.5)	6.81 a	(2.71)	227 b	(42)	3665 ab	(563)	215 c	(25)		
Mainland Greece	9.23 a	(2.62)	14.5 a	(1.3)	5.25 a	(0.58)	218 b	(43)	2744 b	(501)	392 b	(81)		
*p* F-test	NS		0.020		NS		0.043		<0.001		0.012			
	**Na-ex**		**Cu-DTPA**		**Zn-DTPA**		**Fe-DTPA**		**Mn-DTPA**		**B-HW**			
	**(mg kg^−1^)**													
North Aegean Islands	54.0 a	(5.1)	1.87 a	(0.24)	1.15 a	(0.30)	17.2 a	(5.3)	14.1 b	(2.9)	0.74 a	(0.18)		
Crete Island	52.9 a	(4.62)	1.26 b	(0.09)	1.46 a	(0.38)	27.2 a	(5.6)	27.8 ab	(3.4)	0.69 a	(0.08)		
Mainland Greece	31.9 b	(5.9)	1.39 ab	(0.20)	1.26 a	(0.59)	35.0 a	(8.7)	35.5 a	(9.7)	0.60 a	(0.11)		
*p* F-test	<0.001		0.049		NS		NS		NS (0.123)		NS			

## Data Availability

All data are included in this article (and its supplementary information files).

## Supplementary Materials

The following supporting information can be downloaded at https://www.mdpi.com/article/10.3390/biology12040605/s1: Supplementary Material Figure S1: boxplots showing log_10_ concentrations of (a) macro-nutrients (N, P, K, Ca, and Mg) and beneficial element Na, and (b) micro-nutrients (Cu, Zn, Fe, Mn, and B) in subterranean bulb biomass of the studied wild-growing Greek tulip species.
