# Supplementary material for: Adaptability of Wild-Growing Tulips of Greece: Uncovering Relationships between Soil Properties, Rhizosphere Fungal Morphotypes and Nutrient Content Profiles
Source: Biology (Basel). 2023 Apr 16;12(4):605. doi: 10.3390/biology12040605 (PMC10136029; doi:10.3390/biology12040605)
Supplement: Supplementary file 1 [file biology-12-00605-s001.zip › biology-2286435-supplementary.pdf]

biology MDPI

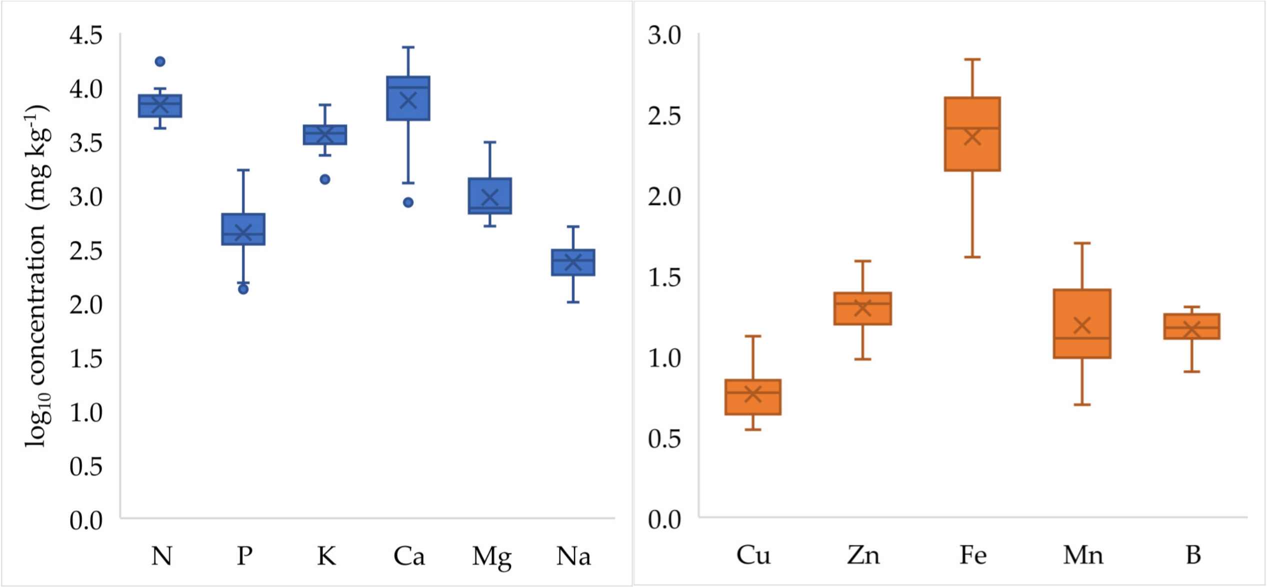

(**a**) (**b**)

Figure S1. Boxplots showing log_{10} concentrations of (**a**) macro-nutrients (N, P, K, Ca, Mg) and beneficial element Na, and (**b**) micro-nutrients (Cu, Zn, Fe, Mn, and B) in subterranean bulbs biomass of the studied wild-growing Greek tulip species.
